# Cross reactivity between cypress pollen and food plants measured by prick test and immunocap

**DOI:** 10.1186/2045-7022-1-S1-P22

**Published:** 2011-08-12

**Authors:** Alejandra Medina Hernandez, Guadalupe Zaldivar-Lelo de Larrea, Carlos Sosa-Ferreyra

**Affiliations:** 1 University of Queretaro, Medicine Faculty, Queretaro, Mexico; 2 Queretaro University, Faculty of Medicine, Queretaro, Mexico; 3 Queretaro University, Biologicals Science faculty, Queretaro, Mexico

## 

The reason for the increase in food allergy are unknow. due the short period of time that has been presented, it is suggested tan enviranmental factors have greater impact than genetics.The geographical conditions of Queretaro and having a large industrial corridor are risk factors for development of allergy problems. In Mexico there are no prevalence studies of food allergies and therefore the most common food allergens. We try to identify common allergen sensitization and determine if there is cross-reactivity between cypress pollend and food plant most commonly consumed in Queretaro city. We performed a correlatin study in patients allergic to cypress pollen to determinate if there is cross reactivit between it and food plants by skin prick test and specific sera titers by immunocap technique. we studied 22 patients, mostly with allergic rhinitis (95.5%), 11 with asthma (50%).10 patients (45.5%) had no first-degree relatives with atopy. The reported heartburn associated wih food intake was 40.1%, while urticaria or discomfort in the oropharynx was 27.2%, 22.7% for edema lips, palate itching or constipation. Using Pearson correlation coefficient was found relationship with apple (0.99), wheat (0.98), celery (0.98), peanut(0.96), melon (0.93), lentil (0.91), tomato (0.91), beans (0.899), avocado (0.87), soybeans (0.82), chickpea (0.81), maize (0.79), pepper (0.79). Although literature reported only association between cypress and tomato, we found relation with other food plants.

